# Assessment of fetal modified myocardial performance index in early‐onset and late‐onset fetal growth restriction

**DOI:** 10.1111/echo.14364

**Published:** 2019-05-22

**Authors:** Lina Zhang, Jijing Han, Na Zhang, Zhen Li, Jingjing Wang, Yinghua Xuan, Karl Oliver Kagan, Qingqing Wu, Lijuan Sun

**Affiliations:** ^1^ Department of Ultrasound Beijing Obstetrics and Gynecology Hospital Capital Medical University Beijing China; ^2^ Department of Women's Health University of Tuebingen Tuebingen Germany

**Keywords:** Doppler flow, fetal growth restriction, modified myocardial performance index, perinatal outcome

## Abstract

**Aim:**

To investigate the changes of modified myocardial performance index (Mod‐MPI) in early‐onset and late‐onset fetal growth restriction (FGR) cases, and its association with adverse perinatal outcome.

**Methods:**

This was a prospective study on 77 early‐onset and 100 late‐onset FGR cases. Hundred normal fetuses were matched as control groups for early‐onset and late‐onset FGR groups, respectively. Mod‐MPI and vessel Doppler parameters including umbilical artery (UA), ductus venosus (DV), and middle cerebral artery (MCA) were measured. Perinatal outcomes were followed up. Mod‐MPI of FGR cases were compared in normal Doppler, abnormal Doppler, and control groups. The association of Mod‐MPI and perinatal outcome was investigated, and further efficacy of Mod‐MPI predicting adverse outcome was studied.

**Results:**

Compared with control groups, both abnormal and normal Doppler groups showed increased Mod‐MPI in early‐onset and late‐onset FGR, respectively. Mod‐MPI had no significant difference between abnormal and normal Doppler groups. Mod‐MPI was associated with adverse outcome in early‐onset FGR (OR = 3.307) and late‐onset FGR (OR = 3.412). The sensitivity and specificity of Mod‐MPI predicting adverse outcome were 60% and 80% when cutoff value was 0.47 in early‐onset FGR. And they were 65% and 70% when cutoff value was 0.50 in late‐onset FGR.

**Conclusion:**

Fetal growth restriction fetuses had increased Mod‐MPI. Mod‐MPI could be used to predict adverse perinatal outcome of FGR fetuses. Mod‐MPI was an effective parameter to supplement vessels’ Doppler parameters in monitoring FGR.

## 1 INTRODUCTION

Fetal growth restriction (FGR) has been a challenging issue in clinical practice. It is significantly related to adverse perinatal outcome. Complications of FGR such as prematurity, respiratory distress syndrome, and necrotizing enterocolitis have increased perinatal mortality and morbidity.^1^, ^2^ Therefore, detecting and monitoring of FGR during pregnancy are critically important, which prompt timely and mode of delivery and improve the outcome of these cases. The intrauterine safety of fetuses has been evaluated extensively by Doppler parameters of umbilical artery (UA), middle cerebral artery (MCA), and ductus venous (DV).^3^, ^4^, ^5^ The changes of these vessels’ Doppler flow reflect the worsening of the cardiovascular condition of fetuses. However, vessels’ Doppler flow spectra of some FGR fetuses remained normal until adverse perinatal outcomes emerged. So, it is essential to find an effective parameter to supplement FGR monitoring.

Myocardial performance index (MPI) is one of the indicators of fetal cardiovascular situations. It is a noninvasive Doppler‐derived indicator that evaluates global myocardial function.^6^ MPI has been proved to be a reliable parameter, not being affected by fetal cardiac ventricular size, fetal heart rate, and geometry.^7^, ^8^ Some studies have demonstrated that FGR cases were associated with prenatal adverse cardiac remodeling.^9^, ^10^ The aim of this study was to investigate the changes of MPI in early‐ and late‐onset FGR cases with different Doppler manifestations and its association with adverse perinatal outcome, to further confirm the clinical value of MPI in detecting and monitoring FGR.

## 2 METHODS

This was a prospective study carried out at the Beijing Obstetrics and Gynecology Hospital, Capital Medical University between October 1, 2016, and October 1, 2017. Fetuses with estimated fetal weight (EFW) < 10th centile for gestational age (GA) according to the formula of Hadlock et al were diagnosed as FGR.^11^, ^12^ These cases were divided into early‐onset (<32 weeks) and late‐onset FGR (≥32 weeks).^13^ Both early‐onset and late‐onset FGR cases were respectively matched with 100 singleton pregnancies with normal maternal and fetal outcome. The study was approved by the institutional review board (IRB) of Beijing Obstetrics and Gynecology Hospital, Capital Medical University (2016‐ky‐071‐01).

### 2.1 Echocardiography

Fetal examination was performed using Samsung WS80A Ultrasound System, with a probe of 1~7 MHz.

**Figure 1:**
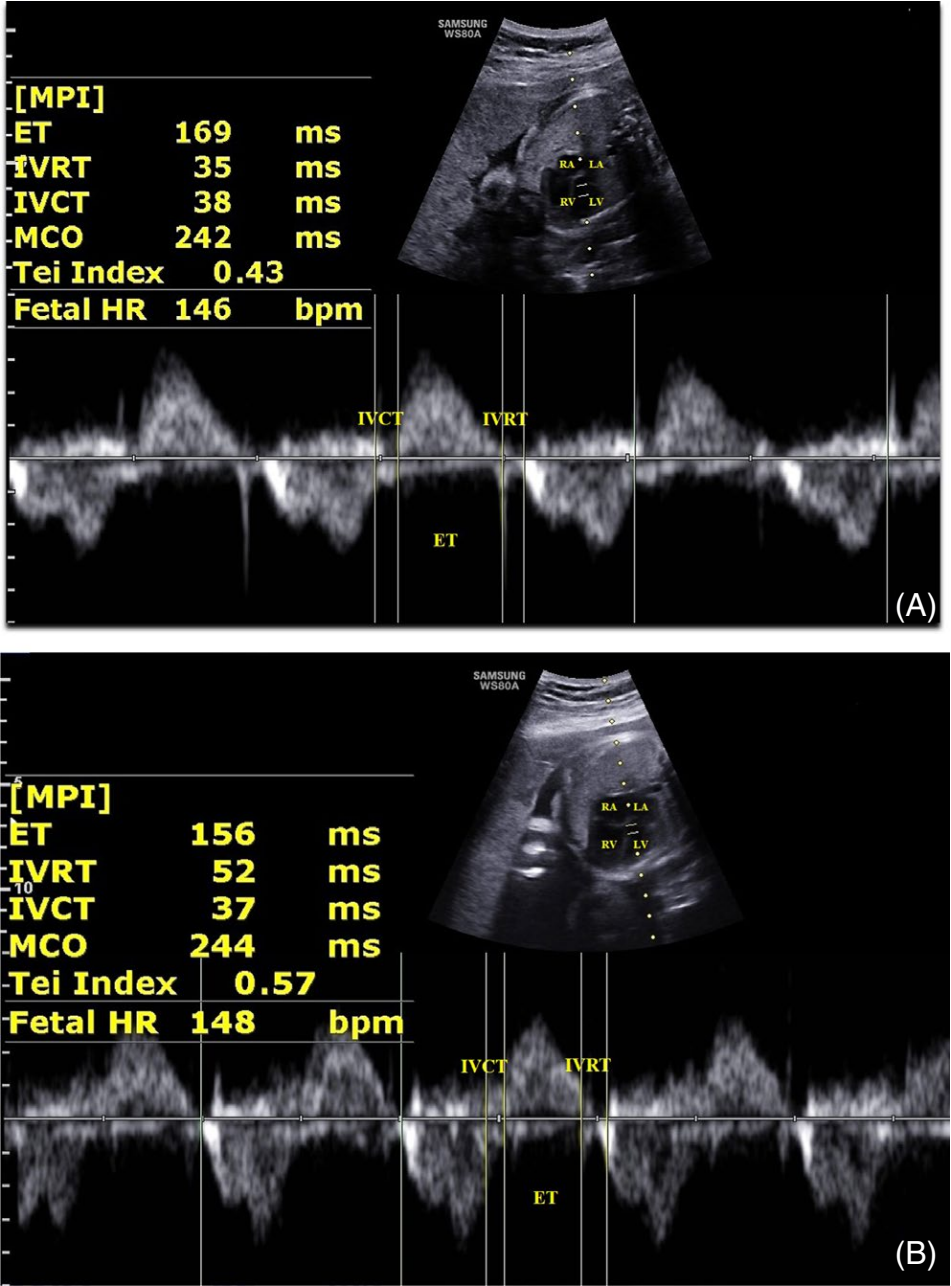
Mod‐MPI measurement in normal fetus (A) and fetal growth restriction fetus (B). ET = ejection time; IVCT = isovolumetric contraction time; IVRT = isovolumetric relaxation time; LA = left atrium; LV = left ventricle; MCO = mitral valve closing and open time; MPI = myocardial performance index; RA = right atrium; RV = right ventricle; Tei Index (Mod‐MPI) = (IVCT + IVRT)/ET; MCO = IVCT + IVRT + ET

The measurement of Mod‐MPI referenced the method recommended by Hernandez‐Andrade et al.^14^ The transverse four‐chamber view with an apical or bottom heart was obtained for MPI Doppler measurement that clearly demonstrated the opening and closing of both the mitral and the aortic valves. The Doppler sample size was adjusted as 3–4 mm to include both internal leaflet of mitral valve (MV) and aortic valve (AV). The Doppler sweep velocity was set as 600 Hz, scale as 55 cm/s, and wall‐motion filter as 100 Hz. Isovolumetric contraction time (IVCT), isovolumetric relaxation time (IVRT), and ejection time (ET) were measured, and MPI was calculated as (IVCT + IVRT)/ET by ultrasound system automatically (Figure 1).

### 2.2 Vessel Doppler ultrasound examination

In each case, routine scanning was performed and referenced practice guideline.^15^

We measured the standard Doppler parameters including the UA pulsatility index (PI), DV PI, MCA PI, and cerebroplacental ratio (CPR, MCA PI/UA PI) according to the ISUOG Practice Guidelines.^16^ Abnormal Doppler flow was considered with one or more of the following conditions: UA PI ＞ 95th centile for GA or absent to reversed UA end‐diastolic flow, DV PI ＞ 95th centile for GA or absent to reversed “A”‐wave, MCA < 5th centile for GA, and CPR < 5th centile for GA.^17^, ^18^, ^19^

### 2.3 Follow‐up

The perinatal management including timely and mode of delivery was determined by the obstetrician in charge. Birth GA, birth weight, delivery mode, Apgar score, neonatal intensive care unit (NICU) admission, and perinatal outcomes were recorded in each case. Adverse perinatal outcomes included stillbirth, neonatal death, and neonates with one of the following serious conditions: Apgar score < 7, neonatal resuscitation, umbilical cord pH < 7.1, and NICU admission caused by FGR severe complications.

### 2.4 Statistical analysis

The study population was grouped in early‐onset and late‐onset FGR groups. And each group was further divided into normal and abnormal Doppler groups according to whether the fetal vessel Doppler parameters were normal or not. One‐way ANOVA was used to compare Mod‐MPI values of normal Doppler, abnormal Doppler, and control groups. Chi‐square test was used to compare cesarean rates of FGR and control groups, perinatal morbidity, and mortality rates of early‐onset and late‐onset FGR groups. Logistic regression was used to analyze correlation of Mod‐MPI and perinatal outcome. Receiver operating characteristic (ROC) curves were adopted to calculate the sensitivity and specificity of MPI predicting FGR adverse outcome. Statistical analysis of data was performed using SPSS version 23.0. A value of *P *<* *0.05 was considered as statistically significant.

## 3 RESULTS

### 3.1 Patient characteristics and perinatal outcomes

A total of 228 singleton cases diagnosed as FGR were recruited in this study. Of these, 24 cases were lost in follow‐up and 27 cases with birth weight > 2.5 Kg were excluded. Thus, the study population consisted of 177 pregnancies: 77 early‐onset and 100 late‐onset FGR. The clinical data of FGR and control groups are shown in Table 1. There were no significant differences in age, height, weight of pregnant women, and GA of ultrasound examination between FGR and control groups. There were significant differences in birth GA, birth weight, and cesarean section rate between FGR and control groups.

**Table 1:** Clinical characteristics of control and FGR groups

Characteristics	Control groups (100)/early‐onset FGR groups (77)		*P* value	Control groups (100)/late‐onset FGR groups (100)		*P* value
Maternal conditions						
Age (years)	30.4 ± 2.6	30.5 ± 3.2	0.934	30.1 ± 3.3	31.1 ± 3.2	0.258^a^
Height (cm)	159.4 ± 2.0	160.5 ± 2.9	0.213	160.5 ± 3.6	160.6 ± 3.2	0.971^a^
Weight (kg)	65.6 ± 3.2	66.9 ± 1.8	0.162	75.4 ± 2.8	75.8 ± 2.9	0.695^a^
GA (weeks)	27.2 ± 3.9	27.7 ± 2.8	0.399	34.8 ± 2.5	35.3 ± 1.7	0.110^a^
Fetal biometry						
BPD(cm)	6.9 ± 1.2	6.3 ± 0.9	<0.001	8.6 ± 1.0	8.2 ± 0.5	<0.001^a^
HC(cm)	25.4 ± 3.8	23.6 ± 3.2	0.001	31.2 ± 1.6	29.9 ± 1.5	<0.001^a^
AC(cm)	23.1 ± 3.9	21.0 ± 3.5	<0.001	30.4 ± 2.6	28.2 ± 1.9	<0.001^a^
FL(cm)	5.1 ± 0.9	4.5 ± 0.8	<0.001	6.7 ± 0.5	6.3 ± 0.4	<0.001^a^
EFW(g)	1180 ± 519	850 ± 324	<0.001	2501 ± 552	2006 ± 345	<0.001^a^
UGA (weeks)	27.3 ± 4.0	25.1 ± 3.0	<0.001	34.6 ± 2.3	32.3 ± 3.4	<0.001^a^
Delivery conditions						
Cesarean	7 (7%)	51 (66%)	<0.001	6 (6%)	26 (26%)	<0.001^b^
GA at birth (weeks)	39.6 ± 1.6	33.2 ± 3.6	<0.001	39.5 ± 1.8	36.1 ± 3.8	<0.001^a^
Birth weight	3246 ± 437	1998 ± 382	<0.001	3289 ± 425	2183 ± 314	<0.001^a^

Abbreviations: UGA = ultrasound gestational age.

a Independent *t* test.

b Chi‐square test.

Fifty‐two cases had adverse outcomes in early‐onset FGR group, including 9 stillbirth, 3 neonatal death, and 40 neonates with serious conditions. Perinatal complications included 13 hypoxic–ischemic encephalopathy (HIE), 6 intraventricular hemorrhage (IVH), 4 respiratory distress syndrome (RDS), 4 viral infection, 5 pneumonia, 2 necrotizing enterocolitis, 1 bilirubin encephalopathy, 2 polycythemia, 1 bronchopulmonary dysplasia, 1 leukoencephalopathy, and 1 sepsis. Seventeen cases had adverse outcomes in late‐onset FGR group, including 3 stillbirth and 14 neonates with serious conditions. Perinatal complications included 5 HIE, 2 IVH, 3 asphyxia, 1 viral infection, 1 polycythemia, and 2 hypoglycemia. Perinatal outcomes of FGR cases are shown in Table 2. Perinatal morbidity and mortality rates in early‐onset FGR were significantly higher than the rates in late‐onset FGR.

**Table 2:** Perinatal outcomes in early‐onset and late‐onset FGR groups

Perinatal outcome	Early‐onset FGR (77)	Late‐onset FGR (100)	*P* value
Good outcome	25 (32%)	83 (83%)	<0.001^c^
Adverse outcome	52 (68%)	17 (17%)	<0.001^c^
Stillbirth	9 (12%)	3 (3%)	<0.001^c^
Neonatal death	3 (4%)	0	N/C
Serious conditions^a^	40 (52%)	14 (14%)	<0.001^c^
Neonatal resuscitation	5 (6%)	1 (1%)	<0.001^c^
Apgar score < 7	12 (16%)	6 (6%)	<0.001^c^
Umbilical cord pH < 7.1	15 (19%)	7 (7%)	<0.001^c^
NICU admission^b^	28 (36%)	11 (11%)	<0.001^c^

Abbreviations: N/C = not applicable.

a One or more conditions

b Caused by FGR with serious complications.

c Chi‐square test.

### 3.2 Comparison of Mod‐MPI in different groups

There were 21 abnormal and 56 normal Doppler cases in early‐onset FGR group, and 13 abnormal and 87 normal Doppler cases in late‐onset FGR group. Compared with control groups, abnormal and normal Doppler groups had increased Mod‐MPI. However, Mod‐MPI was not significantly different between abnormal and normal Doppler groups (Table 3).

**Table 3:** Description and comparison of Mod‐MPI in different groups

LSD	Groups(M ± SD)	Groups (M ± SD)	*P*‐value
Early‐onset FGR	Control group (0.42 ± 0.04)	Normal Doppler group (0.46 ± 0.07)	<0.001^a^
		Abnormal Doppler group (0.48 ± 0.06)	<0.001^a^
	Normal group	Control group	<0.001^a^
		Abnormal Doppler group	0.073^a^
	Abnormal Doppler group	Control group	<0.001^a^
		Normal Doppler group	0.073^a^
Late‐onset FGR	Control group (0.45 ± 0.05)	Normal Doppler group (0.48 ± 0.05)	<0.001^a^
		Abnormal Doppler group (0.49 ± 0.05)	<0.001^a^
	Normal Doppler group	Control group	<0.001^a^
		Abnormal Doppler group	0.282^a^
	Abnormal Doppler group	Control group	<0.001^a^
		Normal Doppler group	0.282^a^

Abbreviations: LSD = least significant difference; M = mean; SD = standard deviation.

a One‐way ANOVA.

### 3.3 Relationship between Mod‐MPI and perinatal outcome

Mod‐MPI (OR = 3.307, 95% CI 1.425–7.674, *P *=* *0.005) and UA PI (OR = 1.542, 95% CI 1.181–2.013, *P *=* *0.001) were associated with adverse outcome in early‐onset FGR. However, MPI (OR = 3.412, 95% CI 1.179–9.877, *P *=* *0.024) and EFW (OR = 0.996, 95% CI 0.994–0.998, *P *=* *0.001) were associated with adverse outcome in late‐onset FGR (Table 4).

**Table 4:** Parameters associated with perinatal outcome

Parameter	B	*X* ^2^	*P*‐value	OR	95% CL (LL UL)
Early‐onset FGR					
MPI	1.196	7.759	0.005^a^	3.307	1.425 7.674
UA PI	0.433	10.130	0.001^a^	1.542	1.181 2.013
Late‐onset FGR					
MPI	1.227	5.124	0.024^a^	3.412	1.179 9.877
EFW	−0.004	11.027	0.001^a^	0.996	0.994 0.999

Abbreviations: CL = confidence interval; LL = lower confidence interval; OR = odd ratio; UL = upper confidence interval.

a Logistic regression analysis.

### 3.4 Sensitivity and specificity of Mod‐MPI predicting adverse perinatal outcome

**Figure 2:**
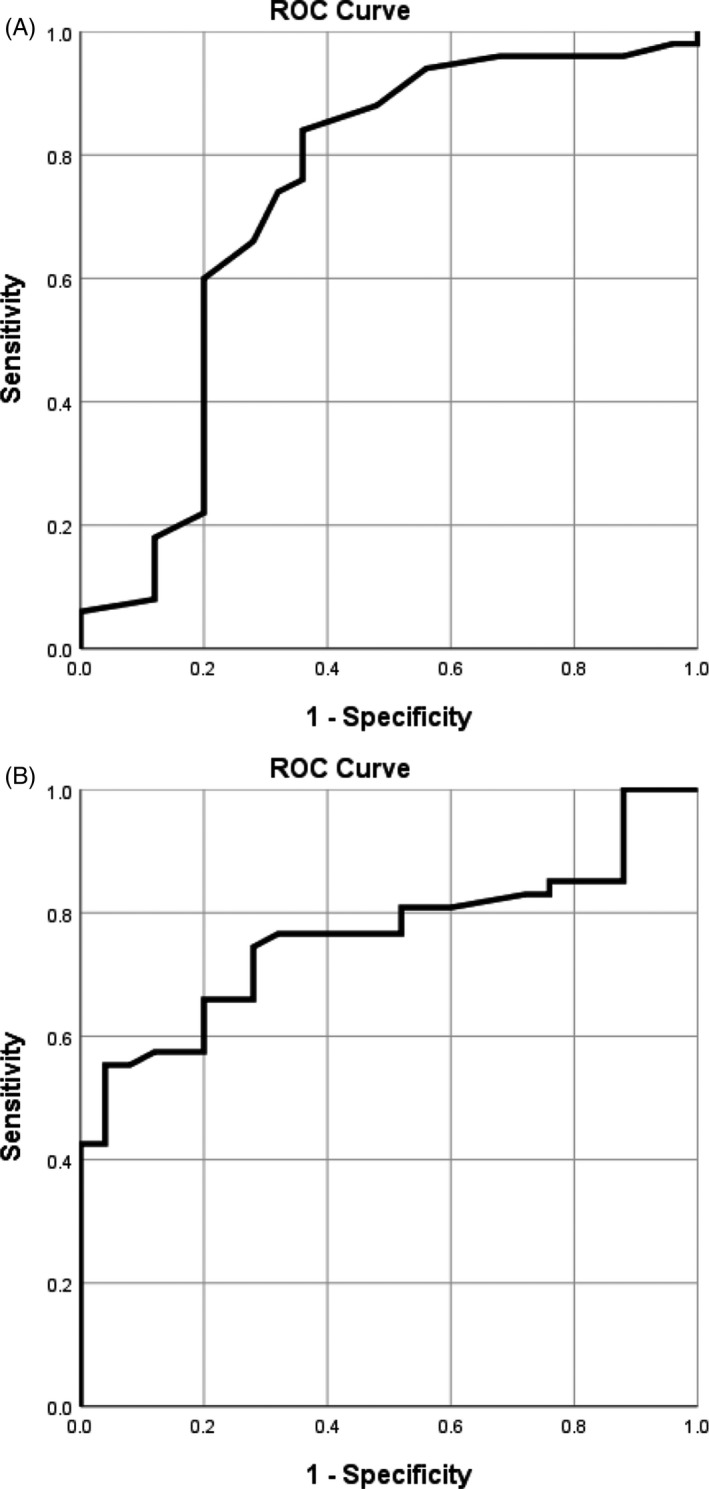
Receiver operating characteristic curve of myocardial performance index (MPI) and umbilical artery (UA) pulsatility index (PI) to predict adverse outcome in the early‐onset fetal growth restriction. The largest AUCs were 0.727 (MPI, A) and 0.772 (UA PI, B)

The largest areas under receiver operating characteristic (ROC) curve of Mod‐MPI and UA PI predicting early‐onset FGR adverse outcome were 0.727 and 0.772, respectively (Figure 2). The sensitivity and specificity of Mod‐MPI predicting adverse outcome were 60% and 80% when cutoff value was 0.47. The sensitivity and specificity of UA PI predicting adverse outcome were 66% and 80% when the cutoff value was 1.10. The sensitivity and specificity of combining Mod‐MPI and UA PI predicting adverse outcome were 86% and 96%. However, the largest AUG of Mod‐MPI predicting late‐onset FGR adverse pregnancy outcome was 0.671. The sensitivity and specificity of Mod‐MPI predicting adverse outcome were 65% and 70% when cutoff value was 0.50.

## 4 DISCUSSION

Fetal growth restriction is one of the most common obstetric conditions, which is associated with increased perinatal mortality and morbidity.^20^ FGR implies that the fetus fails to meet its natural growth potential. Generally, fetuses with EFW < 10th centile for the corresponding gestational week are classified as FGR.^11^, ^12^, ^21^ FGR is divided into early‐onset and late‐onset based on different pathological mechanisms.^13^ The pathological basis of early‐onset FGR is the reduction of the villous vascular area and impaired trophoblastic invasion, resulting in massive lesions of the placenta.^22^, ^23^ However, late‐onset FGR could be diffusion failure from placental maladaptation. Early‐onset FGR is more severe condition; as shown in our study, perinatal morbidity and mortality rates of early‐onset FGR were significantly higher than that of late‐onset FGR. So, we studied MPI in early‐onset and late‐onset groups respectively to exclude potential confounding effects of different pathological mechanisms.

Fetal growth restriction fetus had a decreased and impaired cardiac function probably because of cardiomyocyte growth disruption, which is caused by reduced oxygen and nutrients supply, increasing placental resistance and chronic cardiac afterload.^24^ The application of MPI in FGR has been controversial. Pérez‐Cruz et al^25^ indicated that MPI can be used as a reliable indicator for clinical evaluation of FGR. Nassr et al^26^ showed that MPI was a potentially useful tool which was crucial in classifying FGR pregnancies and predicting neonatal outcome. However, Henry et al^27^ monitored 38 early‐onset and 14 late‐onset FGR fetuses and concluded that MPI was not of clinical value in assessment and management of SGA/FGR fetuses. Pacheco et al^28^ monitored 24 appropriate growth fetuses, 30 fetuses with EFW between the 3rd and 10th centiles, and 22 fetuses with EFW < 3rd centile and also showed that MPI was not significantly different between fetuses with appropriate GA and those with growth restriction. The negative conclusions of the latter two studies might be due to different grouping criteria, different gestational weeks, and small number of cases. In our study, we monitored 77 early and 100 late‐onset FGR fetuses. Our results showed that MPI was significantly increased in FGR fetuses, indicating that MPI was a reliable indicator for monitoring FGR. We further divided FGR into normal and abnormal Doppler groups to study the timing relationship between MPI change and vessel Doppler change. Both normal and abnormal Doppler groups had higher MPI than control groups. There was no significant difference of MPI between abnormal and normal Doppler groups, which implied that impairment of myocardial function might occur earlier than hemodynamic changes.

There are few studies concerning on using MPI to predict perinatal outcome in early‐onset and late‐onset FGR. To further investigate the clinical value of MPI, we used logistic regression to analyze several parameters affecting perinatal outcomes. The results have shown that MPI and UA PI were associated with perinatal outcome in early‐onset FGR. MPI and EFW were associated with perinatal outcome in late‐onset FGR. It is remarkable that only MPI was related to adverse outcome not only in early‐onset but also in late‐onset FGR fetuses in this prospective cohort. We further examined the efficacy of MPI predicting adverse outcome, which showed quite satisfactory sensitivity and specificity. We have found the sensitivity of combining UA PI and MPI predicting adverse outcome was 86% in early‐onset FGR. It seemed that two indicators worked better than single parameter in early‐onset FGR. Moreover, establishment of the cutoff values could assist screening cases with higher risk of adverse outcome.

### 4.1 Limitations

Though we recruited enough FGR cases as possible, our results need confirmation by studies with larger number of cases.

## 5 CONCLUSION

Fetal growth restriction fetuses had increased Mod‐MPI. Mod‐MPI could be used to predict adverse perinatal outcome of FGR fetuses. Mod‐MPI was an effective parameter to supplement vessels’ Doppler parameters in monitoring FGR fetuses.
